# Efficacy and Safety of Perfluorohexyloctane in Evaporative Dry Eye Disease Associated With Meibomian Gland Dysfunction: A Systematic Review and Meta-Analysis of Randomized Controlled Trials

**DOI:** 10.7759/cureus.67920

**Published:** 2024-08-27

**Authors:** Jaime Guedes, Larissa C Hespanhol, Marcos A A Freitas, Caroline C A Balieiro, Maria Eduarda C Souza, Adriano Cypriano Faneli, Hosanna S S Melo, Denisse J Mora-Paez, Bruno M Fontes

**Affiliations:** 1 Ophthalmology, Glaucoma Research Center, Wills Eye Hospital, Philadelphia, USA; 2 Ophthalmology, Opty Group, Rio de Janeiro, BRA; 3 Statistics, Federal University of Campina Grande, Campina Grande, BRA; 4 Medicine, Universidade Estadual da Região Tocantina do Maranhão, Imperatriz, BRA; 5 Medicine, Amazonas State University, Manaus, BRA; 6 Medicine, Maurício de Nassau University Center, Recife, BRA; 7 Medicine, Bahiana School of Medicine and Public Health, Salvador, BRA

**Keywords:** perfluorohexyloctane, nov03, meibomian gland dysfunction, dry eye, meta-analysis

## Abstract

Meibomian gland dysfunction (MGD) is the primary cause of evaporative dry eye disease (DED), which negatively affects the physical and mental quality of life of patients. We performed a meta-analysis of randomized controlled trials (RCTs) comparing perfluorohexyloctane to placebo for MGD in order to identify the best course of treatment for DED in these patients. We followed the Preferred Reporting Items for Systematic reviews and Meta-Analyses (PRISMA) guideline recommendations and prospectively registered the study in PROSPERO (CRD42023442172). The PubMed, Cochrane, and Embase databases were searched for RCTs comparing perfluorohexyloctane to placebo on patients with DED associated with MGD. The statistical analysis was carried out using the “R” software. The mean difference (MD) with 95% CIs was computed using a random-effects model, and p < 0.05 was regarded as statistically significant. The study included 1,814 patients from four RCTs, of whom 972 (53.5%) received perfluorohexyloctane. Patients treated with perfluorohexyloctane had significantly lower total corneal fluorescein staining (tCFS) score (MD -1.09; 95% CI -1.37 to -0.82; p < 0.001; I^2^ = 0%), eye distress Visual Analogue Scale (VAS) (MD -9.69; 95% CI -12.01 to -7.36; p < 0.01; I^2^ = 0%), Ocular Surface Disease Index (OSDI) (MD -5.79; 95% CI -8.22 to -3.36 p < 0.01; I^2^ = 0%), and Eye Burning/Stinging Score (VAS) (MD, -7.16; 95% CI -9.55 to -4.80 p < 0.01; I^2^ = 0%). The meta-analysis results indicate that perfluorohexyloctane was effective and safe in treating evaporative dry eye, reducing tCFS, eye discomfort, OSDI, and burning sensation, despite the included studies only assessing short-term effects and excluding certain patient groups.

## Introduction and background

Dry eye disease (DED) is a multifactorial condition and one of the most common ocular diseases [1-5]. There are two major subtypes of waterless-deficient DED: when lacrimal stashing is reduced, and evaporative DED, which results from the inordinate evaporation of the tear film [6]. The frequency rates of the DED subtypes differed. Estimates suggest that waterless-deficient DED occurs only in 10-15 cases. The considerable level of maturity of DED cases is evaporative or includes evaporative factors [7,8]. Meibomian gland dysfunction (MGD) is the primary cause of DED.

DED patients may experience burning or surcharging sensations, irritation, blankness, and visual disturbances, all of which can have a detrimental impact on their quality of life. Although several therapeutic options are available, such as lipid-based artificial tear substitutes that temporarily replenish the tear film lipid layer, physical treatments like gland expression, warm compresses, thermal palpation, and intense pulsed light therapy, as well as oral antibiotics like doxycycline and azithromycin to reduce inflammation and lower meibum density, these treatments often offer only temporary relief or target specific aspects of the condition [5,9,10].

Perfluorohexyloctane, or NOV03, showed promising results in clinical trials, leading the FDA to approve it in 2023 for the treatment of DED in patients with MGD [11,12]. This is a new nonaqueous ophthalmic drop without preservatives that spreads quickly over the optical face because of its low face pressure [13-15]. The refractive index of NOV03 is similar to that of water, so it does not interfere with vision as much as remedies based on gel or ointment. NOV03 may be superior to existing treatments for DED associated with MGD because it directly addresses the underlying cause by stabilizing the tear film’s lipid layer rather than providing temporary relief. Unlike gels or ointments, NOV03 has a refractive index similar to water, minimizing vision interference. Its preservative-free, nonaqueous formulation also reduces the risk of irritation, making it a safer and more effective option for long-term use. Clinical trials have demonstrated that NOV03 not only alleviates symptoms but also offers more durable and comprehensive relief [11-15].

Thus, we carried out this systematic review and meta-analysis of randomized controlled trials (RCTs) to assess the efficacy and safety of NOV03 for the treatment of DED in patients with MGD.

## Review

Methods

This meta-analysis was performed according to the Preferred Reporting Items for Systematic reviews and Meta-Analyses (PRISMA) guidelines and the recommendations of the Cochrane Collaboration [16,17]. The protocol was registered in the International Prospective Register of Systematic Reviews (PROSPERO) under registration number CRD42023442172.

Eligibility Criteria

Studies that met the following eligibility criteria were included: (1) RCTs; (2) comparing perfluorohexyloctane to placebo; (3) in patients aged ≥18 years with evaporative DED disease associated with MGD; (4) with a follow-up time of at least eight weeks; and (5) reporting any of the clinical outcomes of interest. We excluded overlapping populations, defined as studies with overlapping institutions and recruitment periods; nonrandomized clinical trials; studies in animals; and in vitro experiments. The severity of DED and/or MGD was defined according to the inclusion criteria specified in each study.

Thus, we sought to answer the following question: “Is the use of perfluorohexyloctane safer and more effective than placebo in patients with evaporative dry eye disease associated with meibomian gland dysfunction?”

Information Source

Two authors (LH and CB) independently searched PubMed, Embase, and the Cochrane Library from inception to May 4, 2024. Furthermore, the references from all included studies were also searched manually for any additional studies. Eventual conflicts were resolved by consensus among the authors.

Search Strategy

The following terms were used in this search strategy in August 2023: “(RCT OR randomized OR randomized OR random OR randomly) AND (SHR8058 OR Perfluorohexyloctane OR Perfluorocarbon OR F6H8).” We did not use any publication date or language restrictions in our electronic search for the randomized clinical trials.

Study Selection

We imported search results into the Zotero software (18), and duplicated records were excluded. Two independent authors (LH and CB) applied eligibility criteria to screen the titles and abstracts [18]. Thereafter, the full text of potentially eligible studies was appraised. Any disagreements were resolved by contacting the senior author (BF).

Data Extraction

Two authors (HM and LH) extracted the following data from selected RCTs: (1) ClinicalTrials.gov Identifier; (2) study design; (3) regimen details in the experimental and control arms; (4) number of patients and population allocated for each arm; (5) inclusion and exclusion criteria; (6) time to follow-up; (7) inclusion criteria; and (8) main patient’s baseline characteristics. Additionally, the same authors (HM and LH) collected prespecified baseline characteristics and outcome data and recorded them in Microsoft Excel. Discrepancies were resolved through consensus.

Endpoints and Subgroup Analysis

The primary outcomes of interest were (1) Central Corneal Fluorescein Staining (Central NEI); (2) total corneal fluorescein staining (tCFS) score; and (3) Eye Dryness Score. Other prespecified outcomes included ocular adverse events and Ocular Surface Disease Index (OSDI) scores.

Risk of Bias Assessment

The Cochrane Collaboration tool for assessing the risk of bias in randomized trials (Risk of Bias 2, RoB 2) was used for the quality assessment of individual randomized studies [19]. Two authors independently conducted the risk of bias assessment (LH and MF), and disagreements were resolved by consensus. Each trial was assigned a score of high, low, or unclear risk of bias across five domains: randomization process, deviations from intended interventions, missing outcomes, measurement of outcomes, and selection of reported results. Since the Cochrane guidelines recommend a minimum of 10 studies to adequately assess publication bias using a funnel plot, the analysis was not performed [17,20].

Statistical Analysis

Treatment effects for binary endpoints were compared using pooled RRs or ORs with 95% CIs. Mean differences (MDs) or standardized MDs were used to analyze continuous outcomes. The Cochrane Q-test and I^2^ statistics were used to assess heterogeneity. We determined the between-study heterogeneity based on I^2^ values of 0%, ≤25%, ≤50%, and >50%, indicating no observed, low, moderate, and substantial heterogeneity, respectively. Statistical significance was defined as p-values < 0.05. The Sidik-Jonkman estimator was used to calculate the tau2 variance between studies. We used a random-effects model for all pooled outcomes. For statistical analysis, we used Review Manager (version 5.4) and R (version 4.3.1) software.

Results

Study Selection and Baseline Characteristics

**Figure 1:**
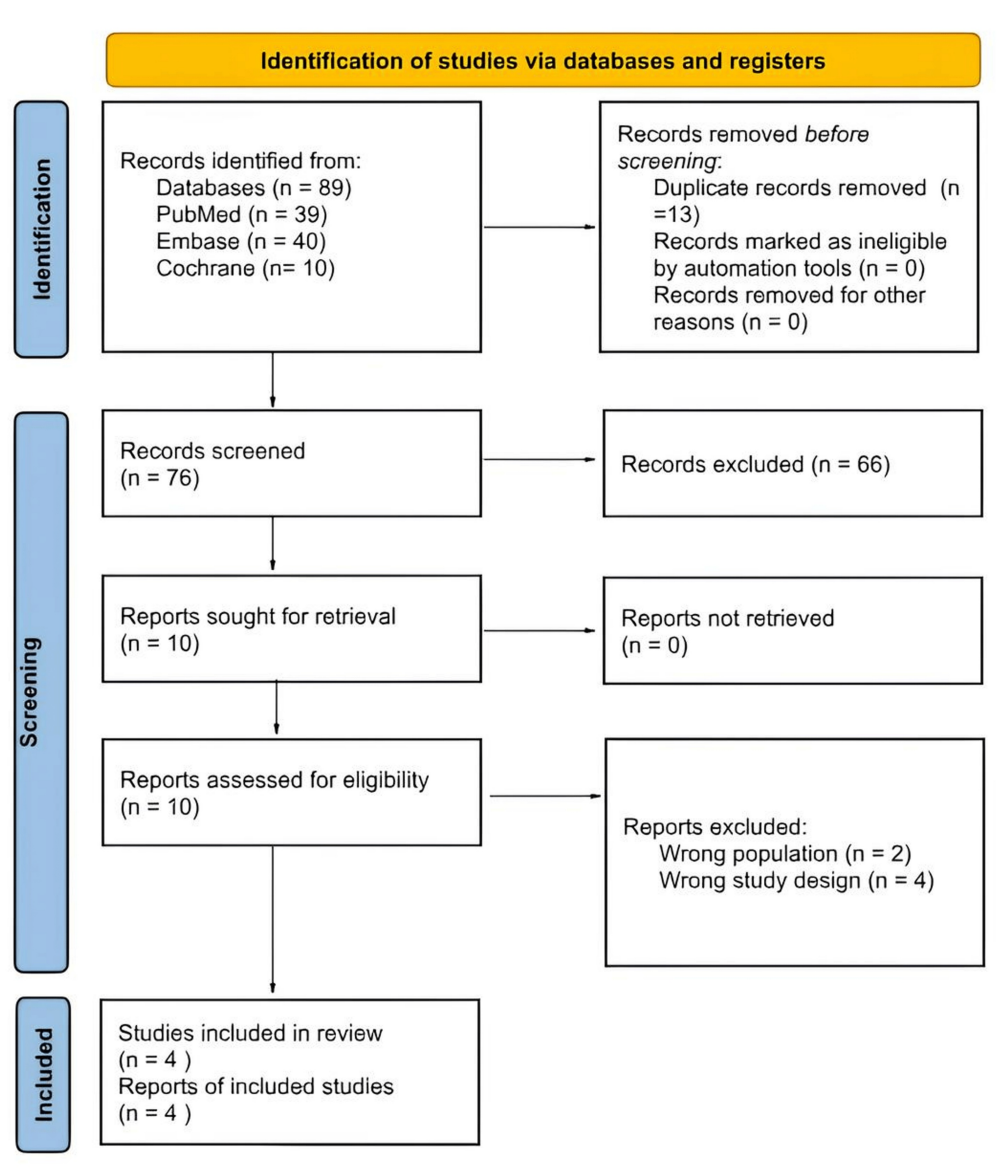
PRISMA flow diagram of the study screening and selection process PRISMA, Preferred Reporting Items for Systematic reviews and Meta-Analyses

Our search identified 118 potential articles; after removing duplicate records and studies with an exclusion criterion based on title/abstract review, four RCTs were included (Figure 1) [11,12,21,22]. Among the patients included in these four studies, 995 (53.5%) received perfluorohexyloctane, while 870 (46.5%) received a placebo (0.6% sodium chloride solution). Three studies had as inclusion categories a complaint of DED symptoms for six months or more at screening [11,12,22]. Another criterion used was reporting a history of DED in both eyes [11,12,21]. One study analyzed NOV03 four times a day (QID) and NOV03 twice a day (BID) [21]. All included studies had eight weeks of follow-up and involved patients aged ≥18 years [11,12,21,22]. The characteristics of the included studies are shown in Table 1.

**Table 1:** Baseline characteristics BID, twice daily; DED, dry eye disease; MGD, meibomian gland disease; NCT, National Clinical Trial number; OSDI, Ocular Surface Disease Index; QID, four times daily; RCT, randomized clinical trial; tCFS, total corneal fluorescein staining

Author	Country	Study design	Time to follow-up	NCT	Number of patients			Age, years		Inclusion criteria
					Perfluorohexyloctane (female)	Placebo (female)	Total	Perfluorohexyloctane (range or SD)	Placebo (range or SD)	
Sheppard et al. (2023) [11] (MOJAVE)	United States	RCT	Eight weeks	NCT04567329	311 (250)	309 (238)	620	53.3 (19-85)	53.8 (20-88)	Briefly, patients ≥18 years of age with a self-reported history of DED in both eyes for ≥6 months were eligible for the study if they met the following key inclusion criteria in ≥1 eye (the same eye) at screening and at randomization: tear film break-up time ≤5 seconds, unanesthetized Schirmer’s tear test I ≥5 mm, total MGD score ≥3, tCFS score ≥4 and ≤11, and OSDI score ≥25
Tauber et al. (2022) [12] (GOBI)	United States	RCT	Eight weeks	NCT04139798	303 (219)	294 (214)	597	60.3 (20-87)	61.6 (19-88)	Adults ≥18 years with a history of DED for ≥6 months, tear film breakup time of ≤5 seconds, Schirmer I test (without anesthesia) score ≥5 mm, MGD score ≥3 (0e15 scale), and tCFS score ≥4 and ≤11
Tauber et al. (2021) [21] (SEECASE)	United States	RCT	Eight weeks	NCT03333057	BID 111 (79) QID 114 (84)	111 (80)	336	QID 53.0 (22-86) BID 54.0 (22-86)	53.8 (19-85)	Adult patients aged >18 years, with a patient-reported history of DED in both eyes, were enrolled in the study if one eye (the same eye) met the following main inclusion criteria at screening and at randomization time: tear film breakup time ≤5 seconds, Schirmer I test ≥5 mm, MGD score ≥3, tCFS score of between 4 and 11, and OSDI score ≥25
Tian et al. (2023) [22]	China	RCT	Eight weeks	NCT05515471	156 (118)	156 (127)	312	45.4 (15.2)	43.7 (15.1)	The diagnosis was based on patient complaints of DED symptoms, an OSDI of 25 or higher, tear film break-up time of 5 seconds or less, Schirmer I test without anesthesia results of 5 mm or more at 5 minutes, a tCFS score of 4 to 11, and an MGD score of 3 or higher

Pooled Analysis of All Studies

**Figure 2:**
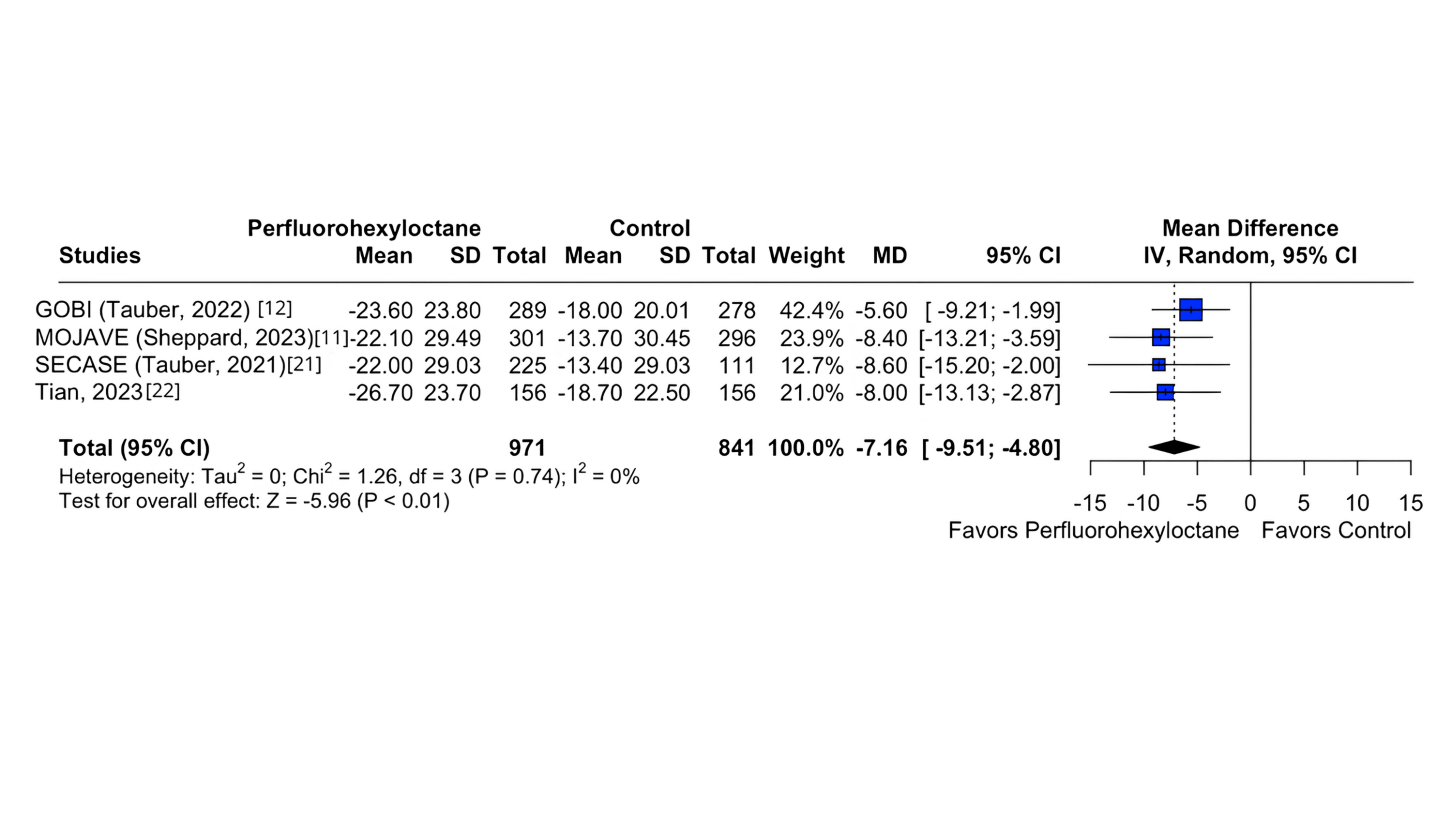
Eye Burning/Stinging Score forest plot This forest plot depicts the change in Eye Burning/Stinging Score (measured by VAS) from baseline to week 8 across different treatment groups. VAS, Visual Analogue Scale

**Figure 3:**
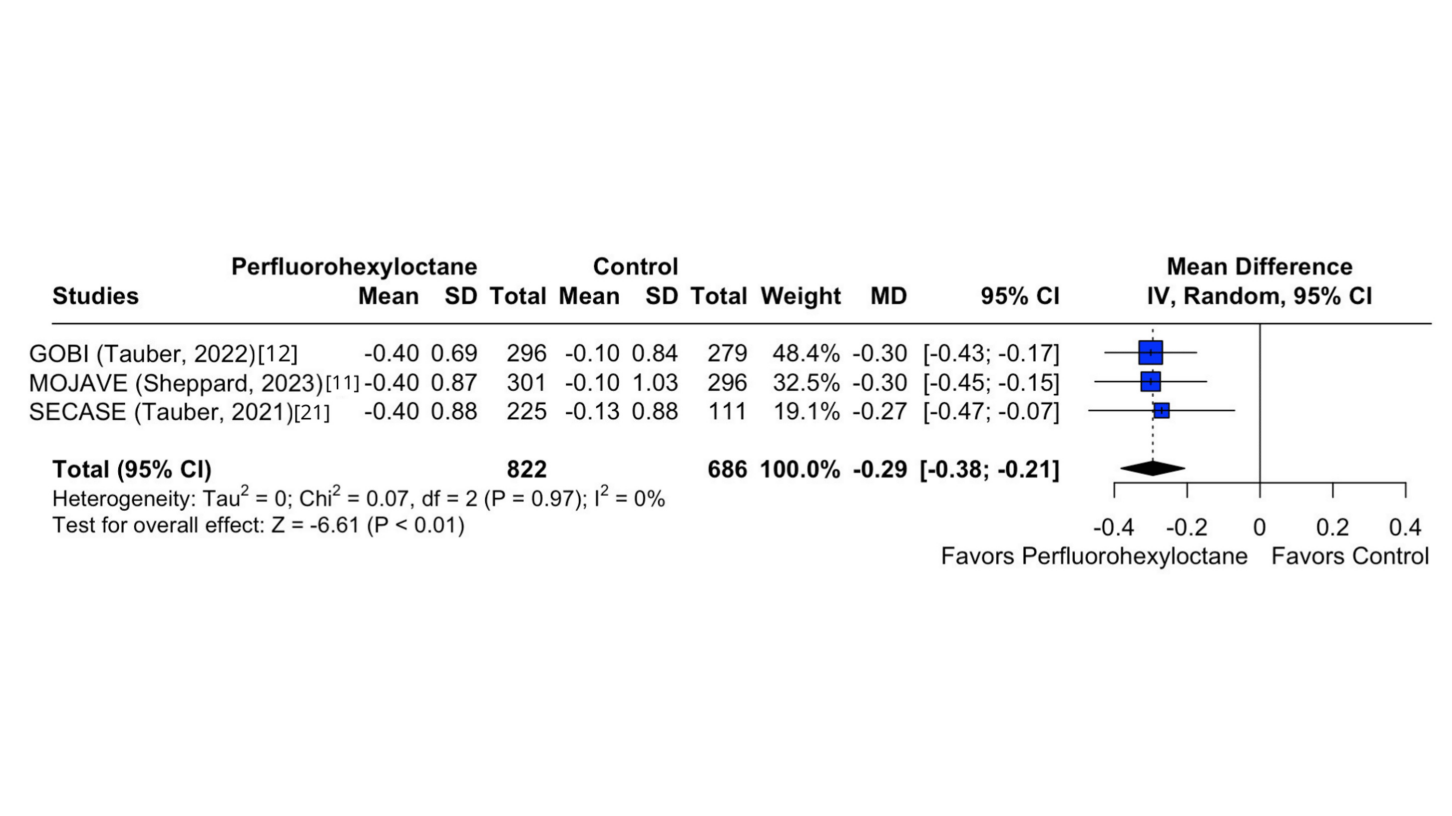
Central NEI forest plot This forest plot shows the change in the Central Corneal Fluorescein Staining Score (NEI scale) from baseline to week 8 across different treatment groups.

**Figure 4:**
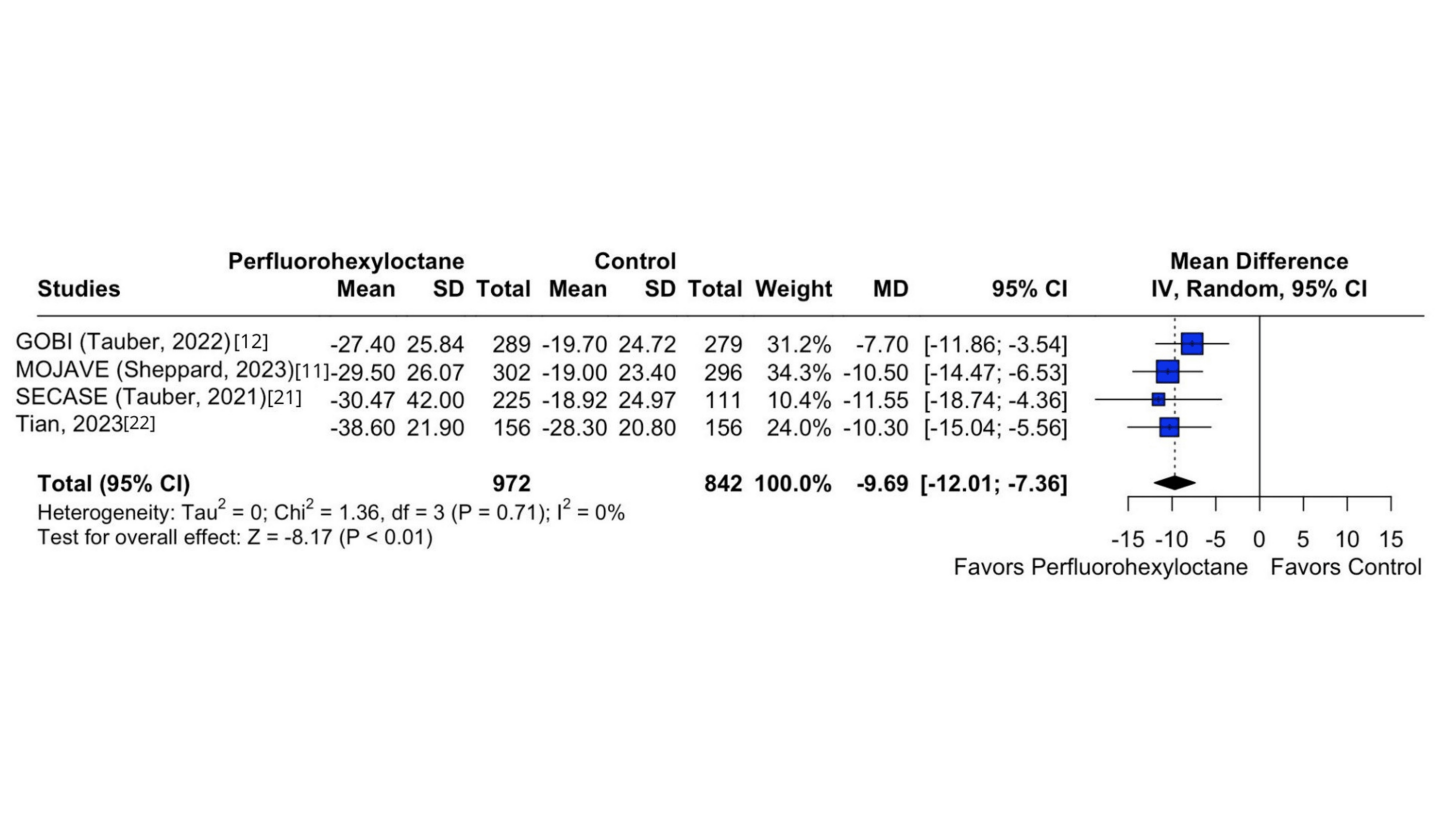
Eye Distress VAS forest plot This forest plot shows the change in Eye Dryness Score (measured by VAS) from baseline to week 8 across different treatment groups. VAS, Visual Analogue Scale

In the investigation of the Eye Burning/Stinging Score, a substantial difference emerged between the perfluorohexyloctane and control groups, as indicated by a statistically significant difference (MD -7.16; 95% CI -9.55 to -4.80; p < 0.01; I^2^ = 0%; Figure 2). Similarly, the Central NEI exhibited a significant disparity between the perfluorohexyloctane and control groups (MD -0.29; 95% CI -0.38 to -0.21; p < 0.01; I^2^ = 0%; Figure 3). In the same way, there was a significant difference in Eye Distress VAS between the perfluorohexyloctane and control groups (MD -9.69; 95% CI -12.01 to -7.36; p < 0.01; I^2^ = 0%; Figure 4). Furthermore, there was a significant difference in tCFS between the perfluorohexyloctane and control groups (MD -1.09; 95% CI -1.37 to -0.82; p < 0.001; I^2^ = 0%).

**Figure 5:**
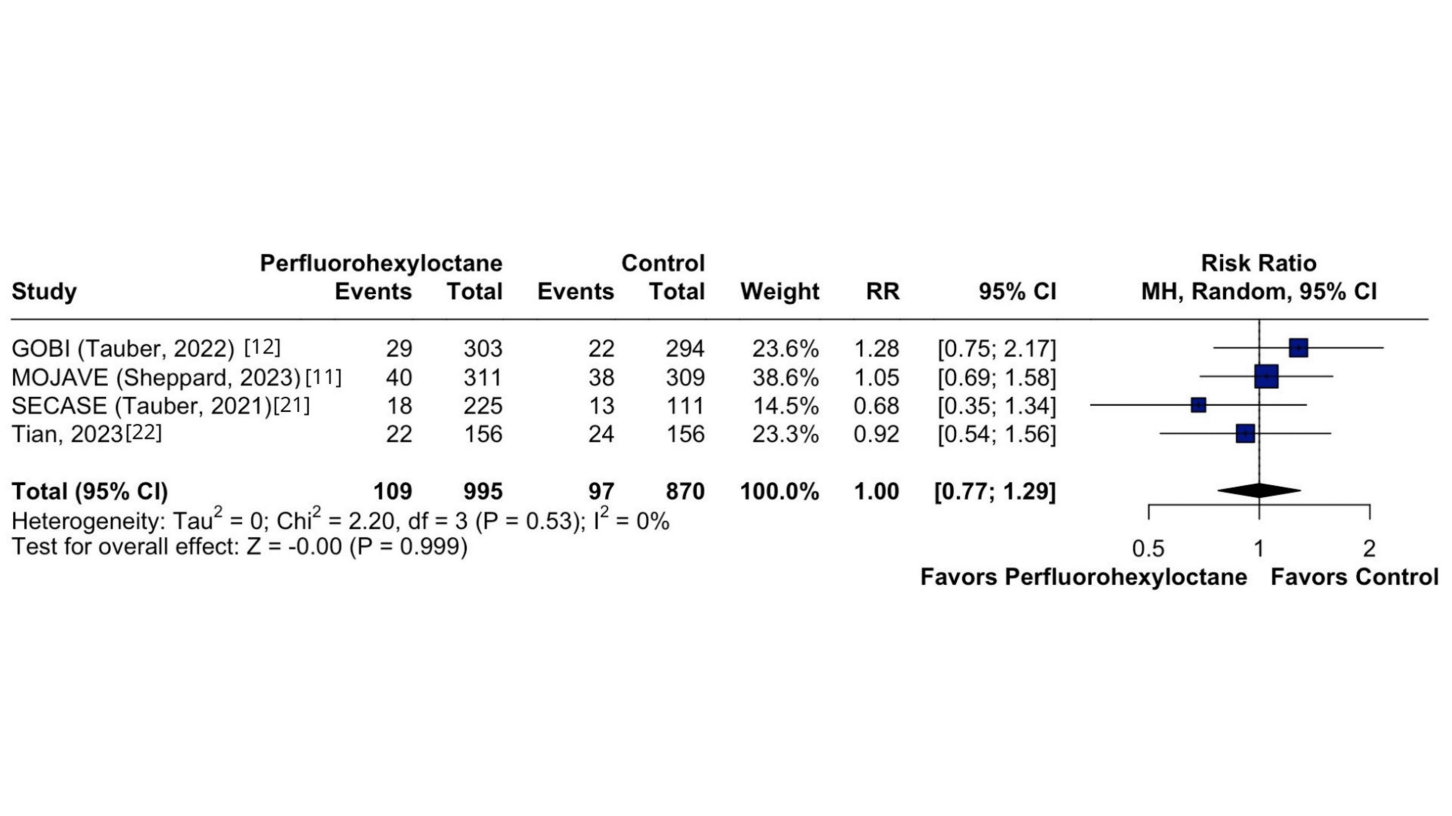
Ocular adverse events forest plot This figure presents a forest plot summarizing the influence of adverse ocular events in different treatment groups.

**Figure 6:**
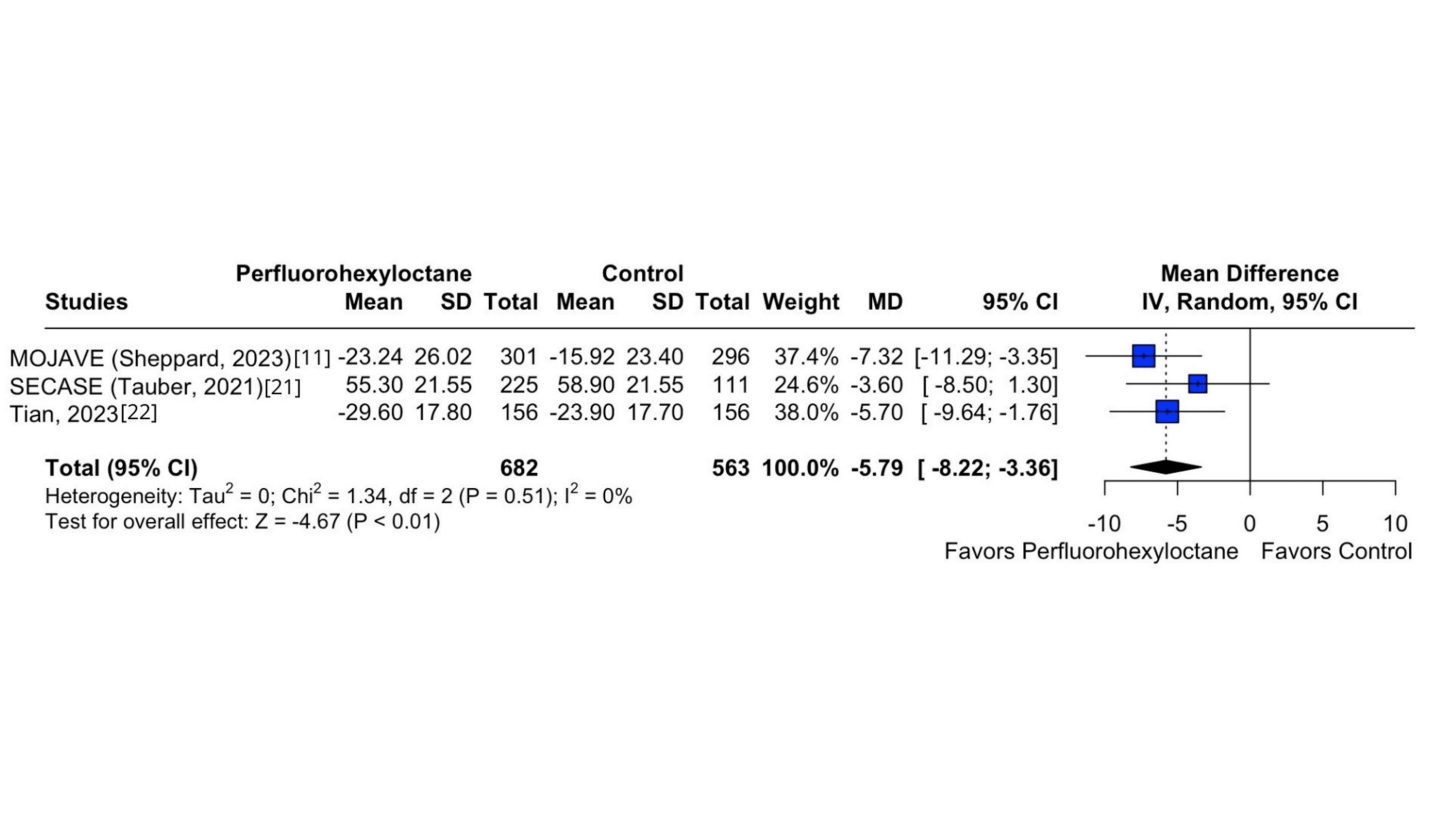
OSDI forest plot This forest plot illustrates the variation in OSDI scores from baseline to follow-up across different treatment groups, displaying effect sizes and CIs to highlight the impact of various interventions on ocular surface health. OSDI, Ocular Surface Disease Index

On the other hand, there was no significant difference in ocular adverse events between the perfluorohexyloctane and control groups (RR 1.00; 95% CI 0.77 to 1.29; p = 0,999; I^2^ = 0%; Figure 5). Conversely, a significant contrast was identified in OSDI between the two groups (MD -5.79; 95% CI -8.22 to -3.36; p < 0.01; I^2^ = 0%; Figure 6).

 *Quality and Evidence Assessment*

**Figure 7:**
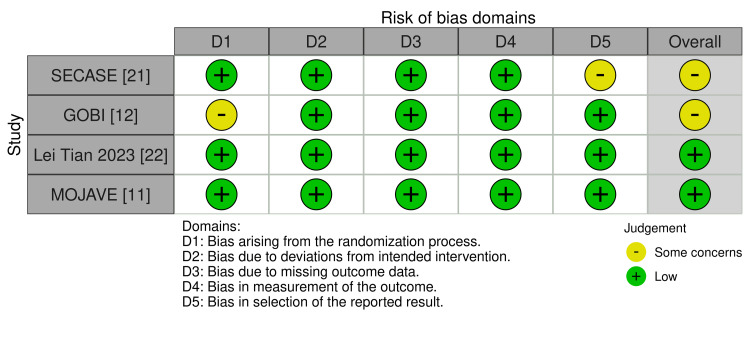
Risk of bias Visual representations, such as summary plots and individual study plots using ROB 2, are widely used tools for assessing the risk of bias in RCTs. RCT, randomized controlled trial; ROB 2, Risk of Bias 2

We assessed the risk of bias in the RCTs using the Cochrane Risk of Bias 2 (RoB 2) tool. All included studies were RCTs, with two studies categorized as having low risk and the remaining two studies categorized with some concerns regarding bias by RoB 2 [16-19]. ROB 2 domain results are presented in Figure 7.

Discussion

Perfluorohexyloctane exhibited substantial clinical and statistical results for all outcomes without any increase in the risk of adverse ophthalmological effects. These findings are particularly significant given the difficulty in demonstrating the benefits of both signs and symptoms of DED. This is due to several factors, such as the limited correlation between dry eye signs and symptoms, high and/or variable placebo treatment responses, and a lack of validated patient outcome instruments for dry eye symptoms [14,23-25].

Recent research has suggested that the Dryness Score (assessed by VAS) may be the most effective tool for capturing patient responses to treatment, as it is the symptom that is most frequently scored highest at baseline and has the lowest variability [14,23-25]. In our meta-analysis, perfluorohexyloctane demonstrated a mean improvement of 7.16 (MD -7.16; 95% CI -9.55 to -4.80; p < 0.01; I^2^ = 0%) and 9.69 (MD -9.69; 95% CI -12.01 to -7.36; p < 0.01; I^2^ = 0%) in the VAS score for Eye Burning/Stinging Score and Eye distress, respectively.

The widely acknowledged view is that the signs and symptoms of DED are often irregular, which hampers the evaluation of treatment effectiveness in clinical trials. This inconsistency has been a major factor contributing to the frequent failures in achieving desired outcomes in terms of both signs and symptoms endpoints [4,14]. Recent research has used OSDI, Central NEI, and tCFS to evaluate the effect of perflurohexyloctane on DED signs and symptomatology [11,12,21,22]. In our meta-analysis, the findings of this study are particularly remarkable, which demonstrate improvement in tCFS, OSDI score, and Central NEI in the perfluorohexyloctane group compared with the control group, which demonstrated a mean improvement of -1.09 (MD -1.09; p < 0.001; I^2^ = 0%), -5.79 (MD -5.79; p < 0.01; I^2^ = 0%), and -0.29 (MD -0.29; p < 0.01; I^2^ = 0%) in the tCFS, OSDI, and Central NEI, respectively. These consistent results demonstrated that perfluorohexyloctane eye drops reduced DED severity and ocular discomfort and improved visual quality and function.

The exact mechanism by which perfluorohexyloctane improves the symptoms of DED related to MGD is not fully understood. However, it is believed to involve the formation of a long-lasting barrier that prevents the evaporation of the aqueous layer beneath the tear film [26,27]. Initially, perfluorohexyloctane’s notably low surface tension enables rapid spreading across the ocular surface. Its amphiphilic properties facilitate the development of supramolecular structures at the lipid-air interface [27-29]. In addition, perflurohexyloctane has the potential to increase tear film spreading and reduce friction during blinking, which could contribute to its positive outcomes [11].

In our study, perfluorohexylcyclohexane emerged as a safe and well-tolerated medication (RR; 1.00; 95% CI: 0.77 to 1.29; p = 0.999; I^2^ = 0%). The exceptional safety profile of perfluorohexylcyclohexane is likely attributable to the fact that eye drops containing this substance are preservative-free and phosphate-free; these substances, commonly found in other formulations, can have adverse effects on the ocular surface and cause blurring, as observed in oil-containing preparations. These account for its enhanced tolerability compared to other approved treatments for DED [30]. The improved tolerability profile compared to currently available therapies is clinically significant, given that up to 60% of patients diagnosed with DED discontinue their treatment within 12 months [31,32].

Limitations

Although the results provided significant insights into the safety and efficacy of perfluorohexyloctane compared to a control for DED associated with MGD, it is crucial to take into consideration the limitations of this study. The limitations of this study include the fact that the studies included in our meta-analysis only followed patients for a short period, which implies that our meta-analysis results have short-term follow-up. Many studies included in this analysis reported follow-up periods of eight weeks. However, long-term outcomes beyond the follow-up period were not assessed. Studies also excluded some patients, such as those who did not wear contact lenses or those who had secondary or severe DED (tCFS score >11). To address the limitations of short follow-up periods in the study, discussing the potential long-term impacts of NOV03 is important. Ongoing research with extended follow-up could clarify whether its benefits are sustained and whether it remains well-tolerated over time. Additionally, expanding on the clinical significance in real-world settings, such as patient adherence and cost-effectiveness, would add depth to the discussion, offering a more comprehensive view of NOV03’s impact in managing DED associated with MGD.

## Conclusions

Our findings suggest that perfluorohexyloctane was effective in the treatment of evaporative dry eye in reducing the tCFS, eye distress, OSDI, and eye burning score when compared to placebo. Furthermore, the use of perfluorohexyloctane eye drops proved to be safe and well-tolerated. Future research could explore NOV03’s long-term effects, broader patient efficacy, and potential in combination therapies, addressing current limitations and expanding its clinical applications.
